# The Impact of Vitamin D Deficiency and Insufficiency on the Outcome of Type 2 Diabetes Mellitus Patients: A Systematic Review

**DOI:** 10.3390/nu15102310

**Published:** 2023-05-15

**Authors:** Zaleha Md Isa, Norizzati Amsah, Norfazilah Ahmad

**Affiliations:** Department of Public Health Medicine, Faculty of Medicine, Universiti Kebangsaan Malaysia, Jalan Yaacob Latif, Bandar Tun Razak, Cheras, Kuala Lumpur 56000, Malaysia; zms@ppukm.ukm.edu.my (Z.M.I.); p114948@siswa.ukm.edu.my (N.A.)

**Keywords:** impact, implication, vitamin D deficiency, vitamin D insufficiency, diabetes mellitus, hyperglycaemia

## Abstract

Vitamin D deficiency and insufficiency are public health concerns that have contributed to multiple negative health outcomes. Recent studies have revealed that vitamin D deficiency and insufficiency influence glycaemic control and the development of diabetes complications. The goal of this systematic review is to summarise the latest evidence on the impact of vitamin D deficiency and insufficiency on the outcome of Type 2 Diabetes Mellitus (T2DM) patients. In this PRISMA-guided systematic review, articles were sourced from three databases, namely, PubMed, Scopus, and Web of Science. The review only included literature published from 2012 until 2022, and 33 eligible studies met the inclusion criteria for this review. The included articles were critically appraised using the Mixed Method Appraisal Tool (MMAT). According to our findings, vitamin D deficiency or insufficiency is associated with mental health status, macrovascular and microvascular complications of T2DM, metabolic syndrome, increased risk of obesity, increased blood pressure, dyslipidaemia, glycaemic control, nerve-related disease, musculoskeletal-related complications, and reduced quality of life. Due to the diverse implications of vitamin D deficiency and insufficiency, screening for vitamin D levels in T2DM patients may be beneficial.

## 1. Introduction

Vitamin D deficiency is a public health concern that involve billions of people and has contributed to multiple health complications [1]. A proportion of the world’s population is at risk for vitamin D deficiency in low and middle-income countries due to inadequate exposure to ultraviolet B (UVB) radiation from sunlight and a lack of foods rich in vitamin D [2]. In recent years, there has been a sharp rise in interest and recent studies have focused on the role of vitamin D in the human body [3].

**Figure 1:**
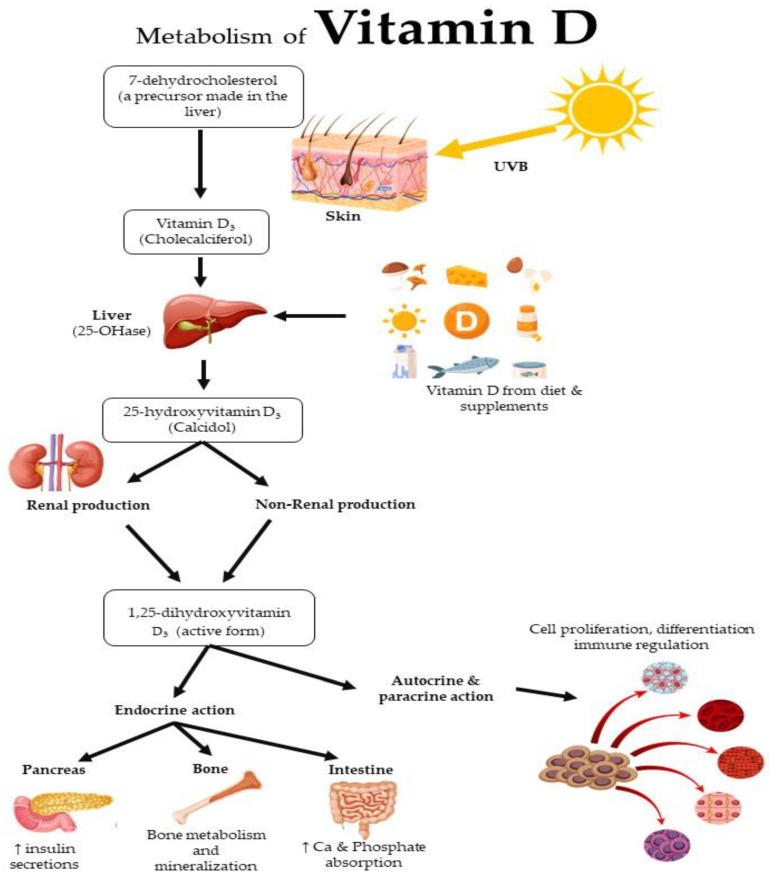
Schematic representation of the vitamin D metabolism, source, and actions.

Type 2 Diabetes Mellitus (T2DM) is one of the most prevalent non-communicable diseases in low-and middle-income countries and is growing at the fastest rate globally in the current decade [4]. In 2017, it was estimated that 451 million people globally have diabetes, and this number is expected to rise to 693 million by 2045 [5]. In recent years, it has been found that T2DM is linked with vitamin D deficiency [6,7,8]. Figure 1 shows a schematic representation of the vitamin D metabolism, source, and actions.

Vitamin D, generally known as calciferol, is a fat-soluble vitamin [9]. There are two main forms of vitamin D: ergocalciferol (vitamin D_2_) and calciferol (vitamin D_3_). Vitamin D_2_ can be synthesised by plants and fungi, while vitamin D_3_ can be synthesised by humans when exposed to UVB from sunlight [2,10]. The major source of vitamin D is exposure to sunlight, which stimulates the production of vitamin D in the skin [11]. When human skin is exposed to UV irradiation, 7-dehydrocholesterol, stored in the skin, is converted to form previtamin D_3_ and subsequently thermoisomerised to vitamin D_3_ [12]. Vitamin D can also be obtained from dietary sources such as dairy products, fatty fish, fortified foods, and supplement intake [13].

Once ingested or synthesised, vitamin D undergoes two hydroxylation steps in the liver and kidneys to reach its biologically active form, 1,25-dihydroxyvitamin D_3_ (1,25(OH)_2_D_3_) [11,14]. Vitamin D is mostly synthesised endogenously when the skin is exposed to ultraviolet B from sunlight, which produces cholecalciferol, and subsequently transported to the liver by the vitamin D binding protein (VDBP). The first hydroxylation occurs in the liver, where 25-hydroxyvitamin is converted to 25-hydroxyvitamin D_3,_ which is the inactive form of vitamin D, with the help of 25 hydroxylase enzymes [9,15]. Subsequently, the vitamin D-binding protein binds to 25-hydroxyvitamin D_3_ and transports it to the kidney for a second hydroxylation. This activation process is carried out by the 1-α 25 hydroxylase enzyme in the kidneys, where 25-hydroxyvitamin D_3_ is converted to 1,25(OH)_2_D_3_, the active form of vitamin D [9].

The final step in the metabolism of vitamin D involves the binding of 1,25(OH)_2_D_3_ to the vitamin D receptor (VDR) in target tissues throughout the body [16]. This active form then binds to vitamin D receptors in target tissues, such as the intestine, bone, endocrine organs, and immune system, to exert its effects [17]. The main actions of vitamin D include promoting calcium and phosphate absorption from the intestine, maintaining bone health, and regulating immune function, cell proliferation, and differentiation [18]. In both experimental and epidemiological studies, vitamin D deficiencies have been linked to decreased insulin release, insulin resistance, and T2DM [19].

Based on the existing literature, epidemiological evidence has shown that vitamin D deficiency is correlated with the onset and progression of many chronic diseases [6]. Furthermore, vitamin D is crucial for a wide variety of extra-skeletal outcomes including immune and inflammatory effects, metabolic syndrome, cancer, and asthma [20]. In different populations, vitamin D deficiency may have different outcomes. For example, among pregnant mothers, vitamin D deficiency increases the risk of gestational diabetes mellitus [21]. The current evidence discusses the outcome of vitamin D deficiency among the general population instead of a specific population such as T2DM patients [22].

There is evidence from multiple studies that vitamin D deficiency may play a significant role in glycaemic control and the development of diabetes complications in T2DM patients [23,24,25,26]. However, it remains controversial and inconclusive. In addition, there is a paucity of reviews that systemically explore the implications of vitamin D deficiency on various outcomes in T2DM patients compared to the general population. Therefore, this review aims to identify available evidence on the impact of vitamin D deficiency and insufficiency on the outcome of T2DM patients.

## 2. Materials and Methods

This review utilised the Preferred Reporting Items for Systematic Reviews and Meta-Analyses (PRISMA) protocol [27]. The protocol for this review was registered with PROSPERO (CRD42022331186). The authors began the review by developing a relevant research question. The process of conducting systematic searching consists of identification, screening, and eligibility processes. During the identification process, three primary databases, namely, Web of Science, Scopus, and PubMed were used. Full original articles were selected to extract relevant information and findings to answer the research question. In terms of the quality of the selected articles, they were screened and evaluated using the Mixed Method Appraisal Tools (MMAT) [28].

### 2.1. Formulation of the Research Question

In this review, the formulation of the research question was based on the PEO (population, exposure of interest, and outcome) concept [29]. This tool is used to assist authors in forming a relevant research question for the systematic review that includes aetiology or risk type of review and aims to identify the association between exposures or risk factors and health outcomes. Thus, the PEO concept, which comprises population, exposure of interest, and the outcome, was recommended. The PEO concept guided the formulation of the research question for this review, which was “What is the impact of vitamin D deficiency and insufficiency on the outcome of T2DM patients?”.

### 2.2. Systematic Searching Strategy

The searching strategy in this review was based on PRISMA flow, which consists of identification, screening, and eligibility stages.

### 2.3. Identification

Relevant keywords using the Medical Subject Headings (MeSH) terms were identified during this stage. Specific search strings were developed using Boolean operators and identified keywords. Search terms were identified as (“effect” OR “impact” OR ”implication”) AND (“vitamin D” OR “25-hydroxyvitamin D OR vitamin D deficiency* OR vitamin D insufficiency*”) AND (“diabetes*” OR “diabetes mellitus” OR “hyperglycaemia”).

All authors independently evaluated the relevance of the titles and abstracts according to the research question. The search string and the systematic search in electronic databases from PubMed, WOS, and SCOPUS based on the keywords were used in the identification process, as shown in Table 1. The systematic literature search was conducted between 31 November 2022 and 31 January 2023. The records were retrieved from the databases and organised on an Excel sheet for screening. Any duplicate articles and titles that deviated from the research question were removed during the process.

### 2.4. Screening by Using Inclusion and Exclusion Criteria

At this point, three authors evaluated the title and abstract of each article for relevance based on the inclusion and exclusion criteria for this review. The inclusion criteria were as follows: (1) publication between 2012 and 2022; (2) full original paper; (3) English language; (4) human studies; and (5) study focused on the research question. The exclusion criteria included conference proceedings, book chapters, editorial letters, and reports. The screening process excluded 2034 articles, and the remaining 39 articles proceeded to full-text retrieval.

### 2.5. Eligibility

A total of 39 full-text articles were successfully retrieved for eligibility. The potential articles identified during the main screening were kept, and the full text was independently reviewed by the two reviewers in detail according to the research question. Any non-related articles were removed. Any disagreement that arose between reviewers was determined by the third reviewer.

### 2.6. Quality Assessment

During the full-text screening, if the article answered the research question, it was critically appraised using the Mixed Method Appraisal Tools [15]. The MMAT evaluates the quality of qualitative, quantitative, and mixed-method studies. It focuses on methodological criteria and includes five core quality criteria for each of the following five categories of study designs: (1) quantitative, (2) qualitative, (3) randomised controlled, (4) non-randomised, and (5) mixed methods. The marks of MMAT for our review were 80–100%, which means the included articles had a good quality appraisal; see the Supplementary Materials.

### 2.7. Data Abstraction and Analysis

Three authors independently extracted the information from the selected studies, including the authors’ names, year, country, study designs, sample size, findings, and the end outcome. Data abstracted from all studies were compiled in a matrix table (Table 2). Three authors reviewed the matrix table for both consistencies and inconsistencies to develop themes and findings for the review. Similar or related information was grouped as one characteristic, and the technique was repeated to form reasonable findings for interpretation.

## 3. Results

### 3.1. Background of the Included Studies

**Figure 2:**
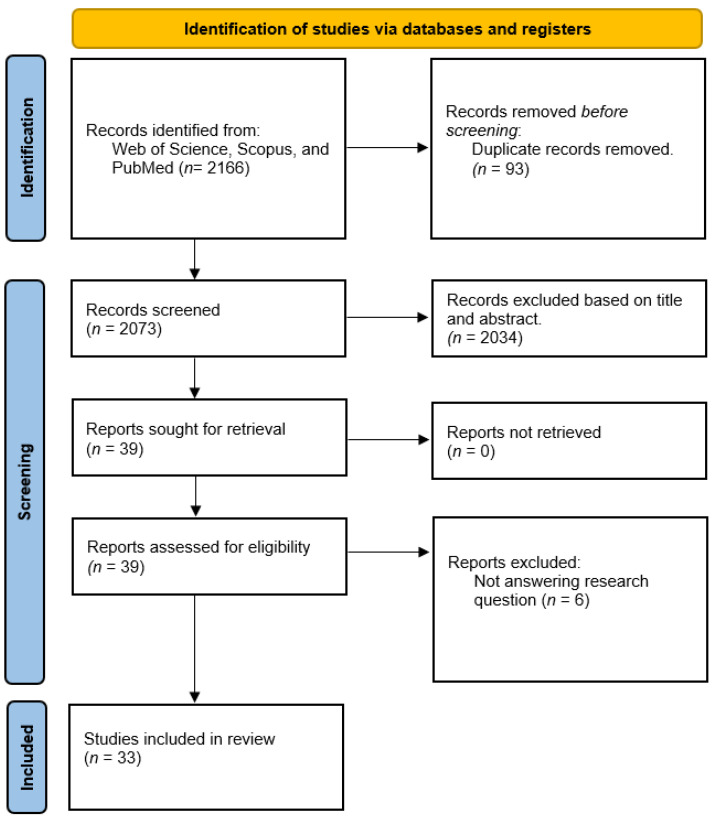
PRISMA flow diagram.

A systematic literature search was conducted and resulted in the retrieval of 2166 records. During the procedure, 93 duplicates and unrelated titles that deviated from the study question were eliminated. Following that, the abstracts were reviewed before the unrelated articles were removed. A total of 39 articles were included in the full-text assessment after rigorous selection and screening. Finally, only 33 articles met the inclusion criteria; they were included in this study, as shown in Figure 2, and the quality appraisal process resumed.

Among the 33 included articles, three were prospective cohorts, 16 were cross-sectional studies, and 14 were case–control studies. The studies were conducted in Egypt, China, Saudi Arabia, the United States of America (USA), India, Greece, Japan, Spain, Iran, Iraq, Sweden, and the United Kingdom. Table 1 summarises the characteristics of the studies, findings, and end outcomes. The selected articles were published between 2012 and 2022.

### 3.2. Outcome Summary of Vitamin D Deficiency among T2DM Patients

All findings were summarised in Table 2. Two studies reported significant associations with mental health and well-being, and two studies showed that vitamin D deficiency has an association with cognitive impairment [42,50]. Twelve studies discussed microvascular complications, which were diabetic retinopathy, neuropathy, diabetic foot ulcers, and nephropathy. Three studies reported a statistically significant association between vitamin D levels and macrovascular complications such as carotid arterial plaque and peripheral arterial disease [44,46,53].

Two studies demonstrated a significant correlation between vitamin D deficiency and increasing body mass index and visceral fat deposition [43,48], while one study demonstrated an association between vitamin D deficiency and metabolic syndrome [55]. Two studies showed an association with hypertension and increased diastolic blood pressure [38,41]. One study showed that vitamin D deficiency increased risk of dyslipidaemia [41]. In terms of glycaemic control, seven studies examined the association of vitamin D deficiency with glycaemic control and two studies shown an inverse association between vitamin D deficiency and insulin levels [30,34,35,48,51,58,60,61,65].

For nerve-related problems, one article showed that vitamin D insufficiency is associated with reduced parasympathetic nerve function [54]. In terms of musculoskeletal-related findings, two studies showed a significant association with bone mass density and one study showed decreased hand grip [37,52,56]. Furthermore, two studies showed that T2DM patients with vitamin D deficiency had reduced quality of life [38,49].

## 4. Discussion

Based on our best knowledge, this is the first review to discuss the impact of vitamin D deficiency and insufficiency on outcomes of T2DM patients. Vitamin D deficiency is a significant health concern that is substantially more prevalent among T2DM patients [34]. The global increase in incidence of vitamin D deficiencies may be due to sedentary lifestyles, junk food, decreased outdoor activities, and less sunlight exposure [66]. Based on our findings, vitamin D deficiency or insufficiency is associated with mental health status, macrovascular and microvascular complications of T2DM, metabolic syndrome, increased risk of obesity, increased blood pressure, dyslipidaemia, poor glycaemic control, nerve-related disease, musculoskeletal-related issues, and reduced quality of life. The findings will be discussed in depth in the following section.

### 4.1. Vitamin D Deficiency and Mental Health

Nutritional factors may play an important role in mental health status among T2DM patients [67]. In this review, two studies reported that vitamin D deficiency was related to mental well-being and poor mental health status, which is depression. Previous studies have shown that vitamin D deficiency is associated with depression and anxiety [68,69]. Vitamin D serum levels were found to be low in T2DM patients with depression [50]. Vitamin D is crucial during serotonin production, and this hormone regulates the feeling and mood of an individual [70]. A case–control study showed that depression patients had lower levels of vitamin D than a healthy group [71]. In addition, a placebo randomised controlled clinical trial study showed that vitamin D supplements are effective in reducing symptoms of mild to moderate levels of depression among T2DM patients [72].

### 4.2. Vitamin D Deficiency and Microvascular Complications

Microvascular complications of T2DM are associated with severe morbidity and mortality, and impact on a high economic burden [73]. According to our review, the findings found that twelve studies showed a significant association between vitamin D deficiency and microvascular complications such as diabetes retinopathy, diabetic neuropathy, diabetic nephropathy, and diabetic foot ulcers. This finding is consistent with a study carried out by Bajaj et al. whereby vitamin D deficiency was found to be associated with an increasing prevalence of microvascular complications, namely, neuropathy, retinopathy, and nephropathy [74]. A meta-analysis demonstrated that vitamin D is able to ameliorate proteinuria and protect from kidney injury among T2DM patients [75].

Other existing evidence suggests that vitamin D deficiency might be a prominent feature of chronic kidney disease, as vitamin D has reno-protective activity [76]. In terms of diabetic neuropathy, vitamin D deficiency may interfere with nociceptor functions by causing diabetic nerve damage, which results in a decrease in the pain threshold compared to the non-diabetic population [77]. Based on our review, low vitamin D level is associated with the increased severity of diabetic retinopathy [36]. It has been postulated that vitamin D deficiency may play a role in the pathogenesis of diabetic retinopathy through its effects on the immune system and angiogenesis [62]. Other than that, from our findings, a study in China showed that vitamin D deficiency was significantly associated with a higher prevalence of diabetic foot ulcers among Chinese T2DM patients [39]. A meta-analysis demonstrated a clear association between vitamin D deficiency and the presence of diabetic foot disease [78].

### 4.3. Vitamin D Deficiency and Macrovascular Complications

From previous literature, vitamin D deficiency has been linked to multiple extra-skeletal effects. Our review demonstrated that vitamin D deficiency is related to macrovascular complications such as peripheral arterial disease and carotid arterial plaque. In our review, two studies discussed the association with peripheral arterial disease, in which it was demonstrated that vitamin D deficiency is associated with endothelial dysfunction as well as arterial stiffness [19,22]. Other than that, Ding et al. found that vitamin D deficiency is related to carotid arterial plaque formation [53].

### 4.4. Vitamin D Deficiency and Metabolic Syndrome

This review also found that metabolic syndrome is one of the effects of vitamin D deficiency among T2DM patients. Metabolic syndrome (MetS) forms a cluster of metabolic dysregulations including insulin resistance, atherogenic dyslipidaemia, central obesity, and hypertension [79]. The recent trend has shown an increased prevalence of MetS over the past few years, as well as an increase in obesity rates, which are associated with poor eating habits and low physical activity [80]. Pan et al. found that among T2DM patients, vitamin D deficiency is one of the risk factors for metabolic syndrome [55]. Vitamin D deficiency may alter insulin secretion and sensitivity, which play a crucial role in the development of MetS [80]. Other than that, a study found that vitamin D supplementation had a positive effect on the treatment of MetS-related disorders, such as lipid profile, insulin resistance, hyperglycaemia, obesity, and hypertension [81].

### 4.5. Vitamin D Deficiency and Risk of Obesity

In this review, two studies showed that vitamin D deficiency may lead to obesity and increased visceral fat accumulation. This is consistent with another study that showed an inverse association between vitamin D levels and obesity [82]. Obesity can be defined as an abnormal or excessive accumulation of fat in the adipose tissue, and body weight more than 20% of the recommended weight [83]. Vitamin D insufficiency leads to decreased insulin, and this condition will activate the lipogenesis mechanism, which will indirectly increase the fat mass [84]. Additionally, vitamin D receptor (VDR) expression in the adipose tissue seems to be associated with vitamin D deficiency in obesity. Vitamin D deficiency will stimulate the activation of the VDR in adipocytes and negatively affect the energy metabolism, which subsequently predisposes to obesity [85]. This is consistent with another study which showed an association between vitamin D level and body mass index in people with T2DM compared to people without T2DM [86].

### 4.6. Vitamin D Deficiency and Blood Pressure

Our review demonstrated that there was a significant inverse relationship between serum concentrations of 25(OH)D and diastolic blood pressure [38]. This condition can be explained with the role of vitamin D as a potent endocrine suppressor of renin biosynthesis and regulator of the renin-angiotensin system (RAS). RAS plays a critical role in the regulation of blood pressure, electrolytes, and plasma volume homeostasis [87]. In addition, a cohort study revealed that vitamin D deficiency is related to dyslipidaemia among T2DM patients [13].

### 4.7. Vitamin D Deficiency and Glycaemic Control

In terms of glycaemic control, our findings demonstrated that vitamin D deficiency is associated with poor glycaemic control among T2DM patients. Seven studies showed a significant relationship between low vitamin D levels and HbA1c levels, and two studies discussed insulin resistance. Instead of the non-calcaemic effect, vitamin D plays a significant role in the hormonal regulation of glucose metabolism [84]. In epidemiological studies, vitamin D deficiency showed a strong relationship with insulin production and insulin resistance among T2DM patients. In our body, vitamin D acts as an epigenetic factor at the transcription level that increases insulin sensitivity [88]. A meta-analysis showed that vitamin D supplementation improves the glycaemic level and insulin sensitivity [89].

### 4.8. Vitamin D Deficiency and Nerve-Related Complications

Previous studies on vitamin D have largely focused on bone health and calcium metabolism. However, there is an increasing interest in the role of vitamin D in the nervous system among T2DM patients. For nerve-related complications, vitamin D deficiency has an influence on nerve function as a study showed that vitamin D insufficiency is associated with reduced parasympathetic nerve fibre function among T2DM [54]. Vitamin D has been linked to the regulation of neurotrophins, which may play a neuroprotective role in the peripheral nerve [90]. The neuroprotective effect of vitamin D is associated with its influence on neurotrophin production and release, neuromediator synthesis, intracellular calcium homeostasis, and the prevention of oxidative damage to the nervous tissue [90].

### 4.9. Vitamin D Deficiency and Musculoskeletal Complications

Guo et al. and Mori et al. found that vitamin D deficiency can reduce bone mineral density among T2DM patients [52,56]. In terms of level and negative impact, vitamin D levels of less than 50 nmol/L or 20 ng/mL may potentially have adverse effects such as fractures and bone fragility, while severe vitamin D deficiency of less than 30 nmol/L or 12 ng/mL increases the risk of death and infection [91]. From our review, vitamin D deficiency led to decreased bone mineral density and contributed to the increased incidence of osteoporosis among T2DM patients, which was more prominent in the femoral neck and total hip of the patients [52].

Vitamin D is essential for calcium absorption and bone mineralisation, which is positively associated with bone mineral density [10]. Recent studies have also shown that vitamin D has an important role in bone formation as it facilitates collagen generation and osteoblasts differentiation [92]. Vitamin D is a steroid hormone that acts by binding to the vitamin D receptors found in many tissues, including skeletal muscle. It has roles in the proliferation and differentiation of muscle cells and skeletal muscle contraction through the calcium-mediated regulation [93]. Hand grip strength is used to estimate total muscle strength. In this review, vitamin D deficiency entails negative consequences on hand grip strength among older T2DM patients [37]. Randomised controlled trials have demonstrated that vitamin D supplementation was able to improve muscle strength [94].

### 4.10. Vitamin D Deficiency and Quality of Life

Quality of life is crucial as it is widely used as an important health outcome measure [95]. In our review, two articles showed a significant relationship between vitamin D deficiency and quality of life among T2DM patients. Based on the findings, there was an association between the serum concentration of 25(OH)D and the physical function component, physical health subscale of quality, social function, and the general health component of quality of life in patients with T2DM compared to the healthy controls [38]. Other studies also showed that vitamin D deficiency is associated with a poor perception of diabetes-specific quality of life [49]. By knowing the relationship between vitamin D levels and quality of life among T2DM patients, screening and intervention may facilitate the management of T2DM patients.

### 4.11. Strengths and Limitations

This review discussed the impact of vitamin D deficiency and insufficiency on the outcome of T2DM patients based on the existing literature by using three databases. However, several limitations are identified in this review. Some studies may have focused on similar topics that were eliminated during the screening process due to the different keywords and titles used by the studies. In addition, only English-language articles were included in this review, and thus a language bias should be considered.

## 5. Conclusions

In conclusion, vitamin D deficiency and insufficiency have multiple impacts on the outcome of T2DM patients. Our findings showed that vitamin D deficiency and insufficiency may influence mental health status, macrovascular and microvascular complications of T2DM, metabolic syndrome, increased risk of obesity, increased blood pressure, dyslipidaemia, glycaemic control, nerve-related disease, musculoskeletal-related complications, and reduced quality of life. Due to the diverse implications of vitamin D deficiency, screening for vitamin D levels in T2DM patients may be beneficial.

## Figures and Tables

**Table 1:** Findings from all the included studies.

Author	Study Location	Study Design	Sample Size	Findings	End Outcome
Darraj et al., 2022 [30]	Saudi Arabia	Cross-sectional	380 patients of T2DM	Vitamin D deficiency is highly prevalent in people with T2DM and is associated with poor glycaemic control.	Glycaemic control
Tang et al., 2022 [31]	China	Case–control study	1721 T2DM patients	A low serum vitamin D level was significantly associated with a higher prevalence of diabetic foot ulcer (DFU) among Chinese patients with T2DM.	Diabetic foot ulcer
Wang et al., 2022 [32]	China	Case–control study	429 T2DM patients	The study confirms that 25-OH-vitamin D is closely correlated with DFU.	Diabetic foot ulcer
Geng et al., 2022 [33]	United Kingdom	Cohort	13,486 individuals (>60 years) with T2DM	Lower serum levels of 25(OH)D were significantly associated with a higher risk of all-cause dementia, Alzheimer’s disease, and vascular dementia.	Cognitive impairment
Bhat et al., 2021 [34]	India	Case–control study	108 cases of T2DM and 101 healthy controls	There is an inverse association between vitamin D and glycaemic control in T2DM patients.	Glycaemic control
Salih et al., 2021 [35]	Iraq	Case–control	Total number of 310 participants were included, 155 with T2DM and 155 patients without T2DM	The serum 25(OH)D levels were significantly lower in patients with poor glycaemic control compared to those with adequate glycaemic control and in individuals with diabetes duration of more than 5 years.	Glycaemic control
Assy et al., 2021 [36]	Egypt	Case–control	80 T2DM patients	There is an inverse relationship between vitamin D levels and neuropathy scores.	Diabetic neuropathy
Mendoza et al., 2021 [37]	Mexico	Cross-sectional study	A total of 116 women ≥ 60 years old with T2DM	The findings showed a relationship between a low circulating level of vitamin D and a decrease in handgrip strength.	Decrease in handgrip strength
Aghamohammadzadeh et al., 2020 [38]	Iran	Case–control	80 overweight/obese subjects with T2DM, and 77 healthy subjects	There was an inverse association between serum concentrations of 25-hydroxyvitamin D and diastolic blood pressure and the physical health subscale of quality of life in individuals with T2DM.	Blood pressure, quality of life (physical health subscale)
Xiao et al., 2020 [39]	China	Cross-sectional	4284 Chinese patients with T2DM	The present study firstly indicated that VDD was significantly associated with a higher prevalence of DFU among Chinese T2DM patients.	Diabetic foot ulcer
Dai et al., 2020 [40]	China	Case–control	51 patients of T2DM	Low serum 25-OH-vitamin D level was associated with DFU in T2DM patients.	Diabetic foot ulcer
Ahmed et al., 2020 [41]	Qatar	Cohort study	496 Qatari subjects, 274 with and 222 without T2DM participated in the study	Vitamin D2 was related to hypertension and dyslipidaemia, while vitamin D3 was associated with diabetic retinopathy.	Hypertension, dyslipidaemia, diabetic retinopathy
Samefors et al., 2020 [42]	Sweden	Cohort study	Data of 761 patients aged 55–66 years	The study found an inverse association between 25(OH)D3 and mental health in patients with T2DM at baseline.	Mental health
Liu et al., 2020 [43]	China	Cross-sectional study	128 adult males with T2DM	There was a significant relationship between vitamin D level and visceral fat accumulation in males with T2DM.	Increase in visceral fat accumulation in males
Yang et al., 2019 [44]	China	Case–control	590 T2DM patients	A low serum 25(OH)D concentration is an independent risk factor for lower extremity vascular pathological changes and acts as a prognostic index for lower extremity atherosclerotic disease in T2DM patients.	Lower extremity atherosclerotic disease
Parveen et al., 2019 [45]	India	Case–control study	Total of 88 T2DM	Serum 25(OH)D was significantly associated with cognitive impairment in T2DM patients.	Cognitive impairment
Yuan et al., 2019 [46]	China	Cross-sectional	1018 T2DM patients	In T2DM patients, lower serum vitamin D levels were associated with an increased risk of peripheral arterial disease.	Peripheral arterial disease
Xie et al., 2019 [47]	China	Cross-sectional study	351 patients with T2DM	Vitamin D insufficiency or deficiency was independently associated with diabetic kidney disease in T2DM.	Diabetic kidney disease
Karau et al., 2019 [48]	Kenya	Cross-sectional study	156 T2DM patients	This study found a significant inverse correlation between vitamin D and glycaemic control and body mass index.	Glycaemic control, body mass index
Alcubierre et al., 2018 [49]	Spain	Cross-sectional	A total of 292 T2DM patients	The study shows the association between vitamin D deficiency and diabetes-specific quality of life in patients with T2DM.	Quality of life
Wang et al., 2017 [50]	China	Cross-sectional study	2786 patients with T2DM recruited from a Chinese diabetes registry.	A significant negative association between serum levels of 25 (OH)D and depression in Chinese patients with T2DM.	Mental health
Saif et al., 2017 [51]	Egypt	Case–control	30 male patients with T2DM and 20 healthy controls	There was a significant inverse relationship between serum vitamin D levels and HbA1c in T2DM patients.	Glycaemic control
Guo et al., 2017 [52]	China	Case–control	368 T2DM, and 300 non-diabetic control subjects	The findings showed a positive correlation between the 25(OH)D level and the bone mass density in T2DM patients.	Bone mineral density
Ding et al., 2017 [53]	China	Cross-sectional	210 T2DM patients	Low serum 25(OH)D concentration was significantly associated with carotid atherosclerotic plaque in T2DM.	Carotid atherosclerotic plaque
Raelene et al., 2017 [54]	United States	Cross-sectional	50 individuals with T2DM	The findings showed that 25(OH)D insufficiency is associated with reduced parasympathetic nerve function, particularly in younger persons with T2DM.	Reduced parasympathetic nerve function
Pan et al., 2016 [55]	China	Cross-sectional	270 T2DM patients aged more than 50 years old	Vitamin D deficiency is associated with related metabolic syndrome among T2DM over 50 years old.	Metabolic syndrome
Mori et al., 2015 [56]	Japan	Cross-sectional	170 postmenopausal women with T2DM	There was a significant positive correlation between 25(OH)D and the radial bone mineral density.	Radial bone mineral density
Zoppini et al., 2015 [57]	Italy	Cross-sectional	715 outpatients with T2DM	Among patients with T2DM, lower levels of 25-hydroxyvitamin D are associated with a higher prevalence of microvascular complications (retinopathy and/or nephropathy).	Microvascular complications (retinopathy and/or nephropathy)
Kajbaf et al., 2014 [58]	France	Cross-sectional	542 medical records of patients with T2DM	There is an association between vitamin D and HbA1c.	Glycaemic control
Tiwari et al., 2013 [59]	India	Case–control	125 T2DM patients and 164 control patients	Severe vitamin D deficiency (<25 nmol/L) is a risk factor for diabetic foot infections.	Diabetic foot infection
Gandhe et al., 2013 [60]	India	Case–control	30 T2DM subjects with 30 healthy controls.	There is a significant inverse association between vitamin D status and insulin levels in T2DM patients.	Insulin resistant
Kostoglou Athanassiou et al., 2013 [61]	Greece	Case–control	120 T2DM and 120 controls	Vitamin D levels appeared to be lower in T2DM patients compared to the control group, with vitamin D levels associated with glycaemic control in T2DM patients.	Glycaemic control
Payne et al., 2012 [62]	United States	Cross-sectional study	A total of 221 patients.	Vitamin D may play a role in the pathogenesis of diabetic retinopathy.	Diabetic retinopathy

**Table 2:** Outcome summary of vitamin D deficiency among T2DM.

Outcome of Vitamin D Deficiency among T2DM Patients		Studies
Mental health	Mental health well-being	[42,50]
	Cognitive impairment	[33,45]
Microvascular complications	Diabetes retinopathy	[41,57,62,63,64]
	Diabetic neuropathy	[36,57]
	Diabetic nephropathy	[47]
	Diabetic foot ulcer	[31,32,39,40,59]
Macrovascular complications	Carotid arterial plaque	[53]
	Peripheral arterial disease	[44,46]
Metabolic syndrome	Metabolic syndrome	[55]
Risk of obesity	Increased body mass index	[48]
	Visceral fat accumulation	[43]
Blood pressure	Hypertension	[41]
	Increased diastolic pressure	[38]
Dyslipidaemia	Dyslipidaemia	[41]
Glycaemic control	Poor glycaemic control	[30,34,35,48,51,58,61]
	Insulin resistant	[60,65]
Nerve related	Reduced parasympathetic nerve	[54]
Musculoskeletal related	Bone mineral density	[52,56]
	Decreased handgrip	[37]
Quality of life	Reduced quality of life	[38,49]

## Data Availability

All data can be found in the article.

## Supplementary Materials

The following supporting information can be downloaded at: https://www.mdpi.com/article/10.3390/nu15102310/s1, Table S1: Details of the mixed method appraisal tool (MMAT) assessment. Table S2: PRISMA Checklist. Reference [96] is cited in the Supplementary Materials.
