# Supplementary material for: The Impact of Vitamin D Deficiency and Insufficiency on the Outcome of Type 2 Diabetes Mellitus Patients: A Systematic Review
Source: Nutrients. 2023 May 15;15(10):2310. doi: 10.3390/nu15102310 (PMC10223393; doi:10.3390/nu15102310)
Supplement: Supplementary file 1 [file nutrients-15-02310-s001.zip › nutrients-2381329-supplementary.pdf]

Supplementary Table S1: Details of the mixed method appraisal tool (MMAT) assessment.

No.	Author	Type of study	Score	1.1	1.2	1.3	1.4	1.5	2.1	2.2	2.3	2.4	2.5
				Is the qualitative approach appropriate to answer the research question?	Are the qualitative data collection methods adequate to address the research?	Are the findings adequately derived from the data?	Is the interpretation of results sufficiently substantiated by data?	Is there coherence between qualitative data sources, collection, analysis, and interpretation?	Is the sampling strategy relevant to address the research question?	Is the sample representative of the target population?	Are the measurements appropriate?	Is the risk of non-response bias low?	Is the statistical analysis appropriate to answer the research question?
1	Darraj et al., 2022 [17]	Quantitative	100%						Yes	Yes	Yes	Yes	Yes
2	Tang et al., 2022 [18]	Quantitative	80%						Yes	Yes	Yes	Cannot tell	Yes
3	Wang et al., 2022 [19]	Quantitative	100%						Yes	Yes	Yes	Yes	Yes
4	Geng et al., 2022 [20]	Quantitative	80%						Yes	Yes	Yes	Cannot tell	Yes
5	Bhat et al., 2021 [21]	Quantitative	80%						Yes	Yes	Yes	Cannot tell	Yes
6	Salih et al., 2021 [22]	Quantitative	80%						Yes	Yes	No	Yes	Yes
7	Assy et al., 2021 [23]	Quantitative	80%						Yes	Yes	Yes	Cannot tell	Yes
8	Mendoza et al., 2021 [24]	Quantitative	80%						Yes	Yes	Yes	Cannot tell	Yes
9	Aghamohammadzadeh et al., 2020 [25]	Quantitative	100%						Yes	Yes	Yes	Yes	Yes
10	Xiao et al., 2020 [26]	Quantitative	80%						Yes	Yes	Yes	Cannot tell	Yes

No.	Author	Type of study	Score	1.1	1.2	1.3	1.4	1.5	2.1	2.2	2.3	2.4	2.5
				Is the qualitative approach appropriate to answer the research question?	Are the qualitative data collection methods adequate to address the research?	Are the findings adequately derived from the data?	Is the interpretation of results sufficiently substantiated by data?	Is there coherence between qualitative data sources, collection, analysis, and interpretation?	Is the sampling strategy relevant to address the research question?	Is the sample representative of the target population?	Are the measurements appropriate?	Is the risk of non-response bias low?	Is the statistical analysis appropriate to answer the research question?
11	Dai et al., 2020 [27]	Quantitative	80%						Yes	Yes	Yes	Cannot tell	Yes
12	Ahmed et al., 2020 [28]	Quantitative	80%						Yes	Yes	Yes	Cannot tell	Yes
14	Samefors et al., 2020 [29]	Quantitative	100%						Yes	Yes	Yes	Yes	Yes
15	Liu et al., 2020 [30]	Quantitative	100%						Yes	Yes	Yes	Yes	Yes
16	Yang et al., 2019 [31]	Quantitative	80%						Yes	Yes	Yes	Cannot tell	Yes
17	Parveen et al., 2019 [32]	Quantitative	100%						Yes	Yes	Yes	Yes	Yes
18	Yuan et al., 2019 [33]	Quantitative	80%						Yes	Yes	Yes	Cannot tell	Yes
19	Xie et al., 2019 [34]	Quantitative	80%						Yes	Yes	Yes	Cannot tell	Yes
20	Karau et al., 2019 [35]	Quantitative	80%						Yes	Yes	Yes	Cannot tell	Yes
21	Alcubierre et al., 2018 [36]	Quantitative	80%						Yes	Yes	Yes	Cannot tell	Yes
22	Wang et al., 2017 [37]	Quantitative	80%						Yes	Yes	Yes	Cannot tell	Yes

No.	Author	Type of study	Score	1.1	1.2	1.3	1.4	1.5	2.1	2.2	2.3	2.4	2.5
				Is the qualitative approach appropriate to answer the research question?	Are the qualitative data collection methods adequate to address the research?	Are the findings adequately derived from the data?	Is the interpretation of results sufficiently substantiated by data?	Is there coherence between qualitative data sources, collection, analysis, and interpretation?	Is the sampling strategy relevant to address the research question?	Is the sample representative of the target population?	Are the measurements appropriate?	Is the risk of non-response bias low?	Is the statistical analysis appropriate to answer the research question?
23	Saif et al., 2017 [38]	Quantitative	80%						Yes	Yes	Yes	Cannot tell	Yes
24	Guo et al., 2017 [39]	Quantitative	100%						Yes	Yes	Yes	Yes	Yes
25	Ding et al., 2017 [40]	Quantitative	80%						Yes	Yes	Yes	Cannot tell	Yes
26	Raelene et al., 2017 [41]	Quantitative	80%						Yes	Yes	Yes	Cannot tell	Yes
27	Pan et al., 2016 [42]	Quantitative	80%						Yes	Yes	Yes	Cannot tell	Yes
28	Mori et al., 2015 [43]	Quantitative	100%						Yes	Yes	Yes	Yes	Yes
29	Zoppini et al., 2015 [44]	Quantitative	80%						Yes	Yes	Yes	Cannot tell	Yes
30	Kajbaf et al., 2014 [45]	Quantitative	80%						Yes	Yes	Yes	Cannot tell	Yes
31	Tiwari et al., 2013 [46]	Quantitative	100%						Yes	Yes	Yes	Yes	Yes
32	Gandhe et al., 2013 [47]	Quantitative	100%						Yes	Yes	Yes	Yes	Yes
33	Kostoglou Athanassiou et al., 2013 [48]	Quantitative	100%						Yes	Yes	Yes	Yes	Yes
34	Payne et al., 2012 [49]	Quantitative	80%						Yes	Yes	Yes	Cannot tell	Yes

Supplementary Table S2: PRISMA Checklist.

Section and Topic	Item #	Checklist item	Location where item is reported
**TITLE**			
Title	1	Identify the report as a literature review.	1
**ABSTRACT**			
Abstract	2	Provide a structured summary including, as applicable: background; objectives; data sources; study eligibility criteria, participants, and interventions; study appraisal and synthesis methods; results; limitations; and conclusions and implications of key findings.<br>See the PRISMA 2020 for Abstracts checklist for the complete list.	1
**INTRODUCTION**			
Rationale	3	Describe the rationale for the review in the context of existing knowledge, i.e., what is already known about your topic.	2
Objectives	4	Provide an explicit statement of the objective(s) or question(s) the review addresses with reference to participants, interventions, comparisons, outcomes, and study design (PICOS).	2
**METHODS**			
Eligibility criteria	5	Specify the inclusion and exclusion criteria for the review and how studies were grouped for the syntheses with study characteristics (e.g., PICOS, length of follow-up) and report characteristics (e.g., years considered, language, publication status) used as criteria for eligibility, giving rationale.	3-4
Information sources	6	Specify all databases, registers, websites, organisations, reference lists, and other sources searched or consulted to identify studies. Specify the date when each source was last searched or consulted.	3-4
Search strategy	7	Present the full search strategies for all databases, registers, and websites, including any filters and limits used.	3-4
Selection process	8	State the process for selecting studies (i.e., screening, eligibility).<br>Specify the methods used to decide whether a study met the inclusion criteria of the review, including how many reviewers screened each record and each report retrieved, whether they worked independently and, if applicable, details of automation tools used in the process.	4
Study risk of bias assessment	11	Specify the methods used to assess risk of bias in the included studies, including details of the tool(s) used, how many reviewers assessed each study and whether they worked independently and, if applicable, details of automation tools used in the process.	4
**RESULTS**			
Study selection	16a	Describe the results of the search and selection process, from the number of records identified in the search to the number of studies included in the review, ideally using a flow diagram.	3 (PRISMA flow)
	16b	Cite studies that might appear to meet the inclusion criteria but which were excluded, and explain why they were excluded.	3; the reason for the studies being excluded is mentioned in the PRISMA flow
Study characteristics	17	Cite each included study and present its characteristics (e.g., study size, PICOS, follow-up period).	5-12
Risk of bias in	18	Present assessments of risk of bias for each included study.	4, appendix document

Section and Topic	Item #	Checklist item	Location where item is reported
studies			
Results of individual studies	19	For all outcomes, present, for each study: (a) summary statistics for each group (where appropriate) and (b) an effect estimate and its precision (e.g., confidence/credible interval), ideally using structured tables or plots.	5-12
**DISCUSSION**			
Discussion	23a	Provide a general interpretation of the results in the context of other evidence.	9-10
	23b	Discuss any limitations of the evidence included in the review.	13
	23c	Discuss any limitations of the review processes used.	13
	23d	Discuss implications of the results for practice, policy, and future research.	15
**OTHER INFORMATION**			
Registration and protocol	24a	Provide registration information for the review, including register name and registration number, or state that the review was not registered.	2
	24b	Indicate where the review protocol can be accessed, or state that a protocol was not prepared.	Not applicable
	24c	Describe and explain any amendments to information provided at registration or in the protocol.	Not applicable
Support	25	Describe sources of financial or non-financial support for the review, and the role of the funders or sponsors in the review.	14
Competing interests	26	Declare any competing interests of review authors.	13
Availability of data, code, and other materials	27	Report which of the following are publicly available and where they can be found: template data collection forms; data extracted from included studies; data used for all analyses; analytic code; and any other materials used in the review.	Not applicable

The checklist has been adapted for KIN 4400 Independent Research Study in Kinesiology at the University of Guelph-Humber. Last updated: Dec 9, 2021

Adapted From: Page MJ, McKenzie JE, Bossuyt PM, Boutron I, Hoffmann TC, Mulrow CD, et al. The PRISMA 2020 statement: an updated guideline for reporting systematic reviews. BMJ 2021;372:n71. doi: 10.1136/bmj.n71.

For more information, visit: http://www.prisma-statement.org/
